# A novel approach to teaching pharmacotherapeutics—feasibility of the learner-centered student-run clinic

**DOI:** 10.1007/s00228-015-1916-x

**Published:** 2015-08-14

**Authors:** Ramon S. Dekker, Tim Schutte, Jelle Tichelaar, Abel Thijs, Michiel A. van Agtmael, Theo P. G. M. de Vries, Milan C. Richir

**Affiliations:** Department of Internal Medicine, Pharmacotherapy Section, VU University Medical Center, room PK 1X74, De Boelelaan 1118, 1081 HZ Amsterdam, The Netherlands; RECIPE (Research and Expertise Center In Pharmacotherapy Education), Amsterdam, The Netherlands; Department of Internal Medicine, VU University Medical Center, Amsterdam, The Netherlands

**Keywords:** Context-based learning, Student-run clinic, Medical education, Pharmacotherapy

## Abstract

**ᅟ:**

Medical students should be better prepared for their future role as prescribers. A new educational concept to achieve this is learning by doing. This encompasses legitimate, context-based training and gives students responsibility as early as possible in their medical education. Student-run clinics (SRCs) are an example of this concept.

**Aim:**

Describe the development of a new SRC for insured patients, primarily focused on medical (pharmacotherapy) education, the learner-centered student-run clinic (LC-SRC), and its feasibility.

**Methods:**

Teams each comprising of three students (first, third, and fifth year) performed consultations including proposing management plans, all under the supervision of an internist. Patients were voluntary selected from the internal medicine outpatient clinic for follow-up in the LC-SRC. Feasibility was evaluated using a set of questionnaires for patients, supervisors, and students.

**Results:**

In total, 31 consultations were conducted; 31 students and 4 clinical specialists participated. A pharmacotherapeutic treatment plan was drawn up in 33 % of the consultations. *Patients* were content with the care provided and rated the consultation with a 7.9 (SD 1.21) (1(min)-10(max)). *Supervisors* regarded LC-SRC safe for patients with guaranteed quality of care. They found the LC-SRC a valuable tool in medical education although it was time-consuming. *Students* appreciated their (new) responsibility for patient care and considered the LC-SRC a very valuable extracurricular activity.

**Discussion:**

The LC-SRC is feasible, and all participants considered it to be a valuable educational activity. It offers students the opportunity to learn in a real interprofessional and longitudinal setting for their future role as prescriber in clinical practice. The benefits and learner effects need to be investigated in a larger study with a longer follow-up.

**Electronic supplementary material:**

The online version of this article (doi:10.1007/s00228-015-1916-x) contains supplementary material, which is available to authorized users.

## Introduction

Junior medical doctors do not feel well prepared for their role as prescriber [1], which is reflected by the number of prescribing errors made by junior doctors [2]. A plausible explanation for a part of this uncertainty and these errors is the gap between the passive role of medical students and their active role as (junior) medical doctors [3].

Learning in a setting similar to the setting of the future profession is called context-based learning (CBL) and relies on four basic principles: setting, repetition, feedback, and responsibility for learning [4, 5]. Learning is an active process, with each student developing his/her own knowledge network built on experience [5, 6]. This experience can be based on fictional cases, patient demonstrations, or observing a consultation. In addition to context learning, the timing of clinical experience is important and experiences should be real and legitimate for optimal learning effects and involvement [7–9]. Learning through service in the (future) workplace (workplace learning) is as old as medicine itself; however, it does not necessarily contain a specific responsibility for the student [8]. Giving students a feeling of responsibility for patient care makes their clinical experiences more “real” and legitimate [7]. Our study group has previously shown that enrichment of the learning context (responsibility for patient care) might be an important factor to improve the training of rational prescribing skills of medical students [10].

The combination of context/workplace learning, early clinical experiences, and sense of responsibility can be conceptualized as learning by doing. This “learning by doing” is a specific example within the more general experiential learning theory that was first described as “learning through reflection on doing” by Kolb [11].

We think that medical students could be prepared better for their future role as prescribers by learning by doing—receiving legitimate, context-based training, and taking on responsibility as early as possible in their medical education. Student-run clinics (SRCs) are an example of the learning by doing concept [12]. These clinics exist for more than 40 years and are completely run and organized by medical students from their first year onward [13]. In a SRC, medical students prepare and perform diagnostic and therapeutic consultations with real patients. Students value the early training opportunity in SRCs and like participating; however, little is known about the effect of SRC participation on students’ skills and knowledge. The quality of care provided in SRCs is good [12].

Recruiting patients could be a potential challenge to setting up a SRC mainly focused on medical education. SRCs were initially established to provide primary care to homeless, poor, and underserved patients, but in many countries, such as The Netherlands, the proportion of individuals without medical insurance is much lower than that in the USA (<1 % in The Netherlands vs 15 % in the USA) [14, 15]. This could mean that fewer patients would attend such clinics. We postulated that the current SRC format could be redesigned into a new, learner-centered student-run clinic for insured patients and with a primary focus on medical education and the teaching of pharmacotherapeutics. We describe the concept and development of a learner-centered student-run clinic with a view to improving the pharmacotherapy skills of future doctors. Secondly, we investigated whether such clinics are feasible.

## Methods

### Description of the learner-centered student-run clinic

In this pilot, medical students from different study years were jointly responsible for the outpatient consultations, including the pharmacotherapy. They collaborated intensively, together and with other health professionals, such as medical specialists and nurses. In order to provide adequate and non-inferior care, and to meet legal requirements, internists bore ultimate responsibility for the consultations. All consultations took place in the internal medicine outpatient clinic and took place in afternoons when consultation rooms were available. All care provided was reimbursed by health insurance companies, as it is normally in regular care.

Every student team of a first-, third-, and fifth-year student prepared their consultation the week before it took place. They made a plan (including history taking, physical examination, additional investigations, and a treatment plan based on the WHO 6-step method [16], which was discussed with the supervising internist. Besides self-study, the students could attend interdisciplinary discussions (e.g., radiology) and additionally consult nurses, administrative personnel, and medical doctors from different disciplines. As a result, they were involved in interdisciplinary learning all based on the patients they would see in the upcoming week. The definite plan for the consultation was agreed upon with their supervisor before each consultation.

From then on, the students had to actively “do the job,” just like a resident—the supervisor was (just) for support. During the consultation, each student had a specific role and responsibility. The third-year student generally performed the consultation, including history taking and physical examination and discussing diagnosis and treatment, together with the fifth-year student. The first-year student complemented with questions and made annotations for the medical record. The supervising internist attended the beginning or end of each consultation and authorized electronic prescriptions when he/she approved the treatment plan. An eventual follow-up consultation within the learner-centered student-run clinic (LC-SRC) was planned to monitor treatment, when possible with the same students. This practice of longitudinal learning, performing complete consultations including the explicit focus on pharmacotherapy is very different from common practice in regular clerkships, were the focus generally lies on diagnostics instead of (pharmaco)therapeutics [17, 18].

**Fig. 1:**
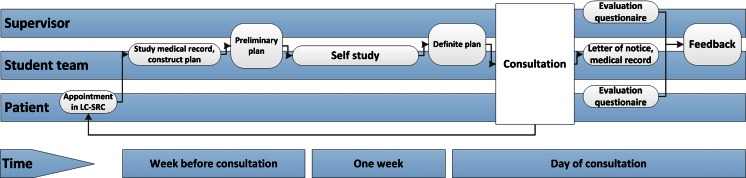
Practice in the LC-SRC

After each consultation, patients could ask the supervisor additional questions and were asked to complete the evaluation questionnaire. Afterwards, the students summarized the consultation in the medical record, wrote a letter to the referring doctor, and the supervisor gave feedback based on the consultation and patient questionnaire (Fig. 1 depicts consultations).

Unique points of this LC-SRC design and concept are (1) Learning by doing as early as possible in medical education (learning in a real context with responsibility for real problems); (2) maximal responsibility; (3) Student-run (organized and run by students); (4) coaching instead of teaching (near-peer teaching within teams and tutoring by supervisor); and (5) Intra- and inter-professional collaboration (working in teams of various experience and disciplines); and (6) Longitudinal learning, with a follow-up of patients.

### Evaluation of feasibility

The LC-SRC was considered feasible if the quality of care was guaranteed and the four following conditions were met:

(1) Patients were satisfied with the care provided; (2) Supervisors considered the LC-SRC feasible; (3) Students considered the LC-SRC feasible; and (4) Sufficient attention was paid to pharmacotherapeutics in the LC-SRC

The feasibility assessment for the three stakeholders (patients, supervisors, and students) was based on assessments in medical education studies [19–21]

1. *Patient* satisfaction with the care provided was measured using a questionnaire about the consultation (student communication skills and professional behavior), readiness to come back to the LC-SRC, and an overall judgment regarding the consultation. The questionnaire was based on a patient evaluation guideline and other evaluations [22–25]
2. *Supervisor* evaluation of feasibility was assessed using a questionnaire on the workability, safety, and quality of patient care in the LC-SRC, readiness to supervise again, additional value for the medical curriculum, suggestions for improvement, and overall opinion of the LC-SRC.
3. *Student* evaluation of feasibility was assessed using a questionnaire on the value of patient contact and responsibility for patient care, quality of organization and supervision, and additional value of the LC-SRC for the medical curriculum. Students could provide feedback and suggestions for improvement. The questionnaire was based on earlier used questionnaires about the value of SRCs [26, 27]
4. *Pharmacotherapeutic* attention was measured as the number of medical treatment plans drawn up, including indications to start or stop treatment and the number of medicines recorded as being used by patients (co-medication). The number of side effects, interactions, and no-longer-indicated drugs recorded were also scored.

#### Population (patients, supervisors and students)

During the first regular consultation at the internal medicine outpatient clinic, patients were asked to attend the LC-SRC for their next consultation, 2–4 weeks later. Patients were not specifically selected (for example on complexity of the case) before they were asked to participate. After each consultation at the LC-SRC, patients were asked to fill in the patient feasibility questionnaire. Four internists with a particular interest in medical education were approached to supervise the LC-SRC. First- and third-year medical students were told about the clinic during a pharmacotherapy lecture and were selected on the basis of a motivation letter and curriculum vitae. Fifth-year medical students who performed their clinical internship in internal medicine in the VUmc were asked to participate in this study, replenished with fifth-year medical students outside their regular clerkship internal medicine.

#### Analysis

All data were imported in SPSS (IBM, version 20.0). Descriptive statistics were used to report the results of the patient, supervisor, and student feasibility surveys. All qualitative feedback from stakeholders was recoded and divided into main and subcategories based on the Grounded Theory from Glaser & Strauss [28, 29]. Two authors (RD and JT) independently read all feedback, derived main and subcategories, and scored the text given in these main and subcategories. These scores were compared, and, if necessary, discrepancies were resolved by a third author (TS).

The institutional review board of the VU University Medical Center reviewed the research protocol. This study did not fall under the scope of the Dutch Medical Research Involving Human Subjects Act (WMO). All stakeholders were informed about the study in advance, gave active verbal and/or written consent, and participated on a voluntary basis.

## Results

### Feasibility assessment by patients

In total, there were 31 consultations with 23 patients with various problems such as osteoporosis, *H. pylori* gastritis, and hypertension/IgA nephropathy. Questionnaires were returned for 29 consultations (94 %) (complete results, see Table 1). Patients considered the students’ professional behavior sufficient-excellent in 26 consultations on a 5-point Likert scale. Twenty-two patients were willing to come back to the LC-SRC. All responding patients agreed the LC-SRC has additional value for medical education. The average score for the consultation was 7.9 (SD 1.21) (scaled 1–10 (minimum-maximum)).

**Table 1:** Results of patient evaluation questionnaire (*n* = 29)

Statements in patient evaluation questionnaire What is your opinion about the...	Scale	Mean (SD)
... introduction and the explanation of the procedure by the students?	1–5	4.2 (0.66)
… feeling of being taken seriously by the students?	1–4	3.6 (0.56)
… information received from the students (possibility to ask questions, answers given to you)	1–5	4.2 (0.79)
… feeling comfortable with the students during this consultation?	1–4	3.6 (0.62)
… professional behavior of the students (in comparison with a medical doctor)?	1–5	3.8 (0.91)
… final assessment of this consultation	1–10	7.9 (1.20)

4-point scale: 1—insufficient, 2—dubious, 3—sufficient, 4—completely; 5-point scale: 1—insufficient, 2—dubious, 3—sufficient, 4—good, 5—excellent; 10-point scale: 1—very poor to 10—excellent

### Feasibility assessment by supervisors

All four supervising internists completed the questionnaire and judged the professional behavior of students as sufficient to excellent (5-point Likert scale). They were all of the opinion that the LC-SRC was safe for patients, that the quality of care was guaranteed, and that the LC-SRC had added value for the students. The workability of the LC-SRC concept was considered dubious by one supervisor, because of the time needed for guidance. All supervisors mentioned this (supervision took 30–60 min per consult); however, all would be willing to supervise in the LC-SRC again. All supervisors reported that the LC-SRC should be continued and pointed out that this pilot has additional value for medical education. It was suggested that residents, rather than internists, could supervise the students to save time. The average score for the LC-SRC was 7.8 (SD 0.5) (scaled 1–10 (minimum-maximum).

### Feasibility assessment by students

Thirty-one medical students participated: 11 first-year, 10 third-year, and 10 fifth-year students. Twenty-nine students returned the questionnaire (Supplemental digital content 1 baseline characteristics). All students felt responsible for the care of patients, valued this responsibility, and thought that working in student teams was a valuable experience. Twenty-five found the LC-SRC well organized, 26 thought it was safe enough to send a relative there, and 27 found the supervision good. Twenty-eight students considered the LC-SRC a valuable addition to the medical curriculum (Table 2, results of the feasibility questionnaire).

**Table 2:** Results of student evaluation questionnaire on feasibility, scored on a 5-point Likert scale; strongly disagree (1), disagree (2), neutral (3), agree (4), and strongly agree (5); (*n* = 29) Full table is available as Supplemental digital content

Statements in student evaluation questionnaire scored on 5-point Likert scale (1–5)	Mean (SD)
I had enough time to prepare for my consultations	4.1 (0.88)
I could easily combine the LC-SRC with the regular curriculum	3.8 (0.97)
I could easily combine the LC-SRC with other activities	3.9 (0.88)
I knew my tasks in the LC-SRC	3.6 (0.82)
The LC-SRC was well organized	4.0 (0.53)
I think patients did not have to wait too long for their consultation	4.0 (1.05)
The LC-SRC ran smoothly	3.6 (0.87)
I would feel happy about sending a relative to the LC-SRC	4.3 (0.75)
I valued seeing patients at the LC-SRC	4.7 (0.47)
I felt responsible for patient care at the LC-SRC	4.7 (0.45)
Real learning in practice at the LC-SRC is more interesting than learning from (fictive) casuistry	4.8 (0.58)
Real learning in practice at the LC-SRC is more instructive than learning from (fictive) casuistry	4.7 (0.60)
I think working in a team is instructive	4.7 (0.47)
I have learned from other student participants	4.6 (0.69)
I think contact with the supervising doctors was good	4.4 (0.63)
I think guidance was good	4.5 (0.63)
I think a LC-SRC would be a valuable addition to the medical curriculum.	4.7 (0.54)

The positive feedback from the students covered all categories. The most positive feedback was for the main category “responsibility and independence,” with “responsibility for patient care” and “thinking about differential diagnosis, additional investigations, and treatment” being the most mentioned subcategories. For example, one student wrote: “I was stimulated to think autonomously about a real patient problem and to make a definite plan.” The most frequently given suggestion for improvement concerned the organization of the SRC, with better planning/communication being the most mentioned subcategory. As example, another student wrote “It would be better to make a complete schedule with planning for all students.”

### Pharmacotherapeutic attention in the LC-SRC

Twenty-one treatment plans were made, and in 11 consultations, more than one pharmacotherapeutic intervention (start/modification/stop) was proposed. In 14 cases, a medicine was prescribed for a (new) diagnosis including lisinopril, *H*. *pylori* triple therapy (pantoprazole, amoxicillin, and clarithromycin), levothyroxine, simvastatin, and colecalciferol with calcium supplements. Patients used a mean of 2.8 (range 0–10) co-medications. In seven cases, a medicine was stopped, three because of side effects, three because it was no longer indicated, and one because of a serious drug interaction/side effect.

Of the 23 patients, 13 could be referred back to the general practitioner or sent to another medical specialist, 7 came to a follow-up appointment at the LC-SRC, and 3 were referred to the regular outpatient clinic or to an internist with a specific subspecialty.

## Discussion

This is the first description and evaluation of an LC-SRC with insured patients in an European healthcare system. Based on the conceptual framework of learning by doing, we developed a novel approach to the acquisition of pharmacotherapeutic knowledge, skills, and competences by medical students. We provided proof of concept regarding the LC-SRC feasibility, since patients and students were satisfied and the supervisors considered the LC-SRC feasible. A short introduction movie of the LC-SRC in our institution is available online (via www.vumc.com/recipe).

### Feasibility, the patient, supervisor, and student perspective

Patients were satisfied with the care provided and were willing to come back. These results are comparable with earlier studies [12, 30, 31]. The focus on learning instead of providing care for uninsured patients seemed to have had no effect on the patient’s satisfaction. Furthermore, even insured patients who had the possibility to choose their healthcare provider were willing to attend student consultations.

The supervisors thought that the LC-SRC was safe for patients and that the quality of care was guaranteed. They considered that the LC-SRC had added value for medical education and all would be willing to supervise in the LC-SRC again. The supervisors spent 30–60 min supervising each consultation, which was long as addition to their “normal” work to do (i.e., supervision of interns and discussion of consultations with residents). To reduce the demand on their time, it might be possible to delegate some aspects of supervision to residents. This supervision could be beneficial to residents, as they gain the opportunity to supervise and get involved in clinical teaching. An earlier study has already demonstrated that SRCs could be a good place to learn (clinical) teaching [12, 32].

The students considered the LC-SRC feasible, they valued the responsibility for patient care, considered working in student teams a valuable experience, and found the LC-SRC a valuable addition to their medical curriculum. Similar results on student satisfaction and perceived improvement in skills/knowledge have been reported previously [12, 27]. Moreover, the students from the different years learned from each other and learned to work together. As SRCs can improve interprofessional learning [33], working in a LC-SRC is an opportunity to learn to work with professionals from different disciplines. In this pilot study, students cooperated with nurses, administrative personnel, and medical doctors from different disciplines, as it is part of the daily outpatient work of medical doctors.

Next to the interprofessional learning opportunities, the LC-SRC concept enables students to follow their patients and initiated (pharmaco)therapy and is therefore an example of longitudinal learning. This concept was previously described within the Harvard Medical School-Cambridge Integrated clerkship and seems to offer significant advantages for learning and professional development for medical students [34].

A concept pharmacotherapeutic treatment plan was drawn up in one third of all consultations. While restrained and balanced therapeutic decision-making is desired, it would be instructive if students were able to draw up more treatment plans to increase their prescribing knowledge and skills. This is in accordance with the theory of context-based learning [4] and the hypothetical model of therapeutic reasoning [35]; Learning by doing in the context of the future profession could stimulate therapeutic script development and evolution [12]. Furthermore, earlier studies have reported good results of pharmacotherapy interventions in SRCs [36, 37]. It would be interesting to start and evaluate specific pharmacotherapy projects, such as polypharmacy checkups.

### Reflections on the pilot—lessons learned

Besides the questionnaire evaluations, we will share some reflections/tips for starting a LC-SRC (Table 3). Most importantly would be to start with an enthusiastic working group of physicians and students. We started with two (then) sixth-year medical students who organized the LC-SRC (RD, TS). In follow-up projects, medical students who had already worked in a LC-SRC could take on the planning and organization. Doing so, a self-sustainable organization is developed where students enroll, gain experience, and take on other, non-medical, responsibilities. SRCs that provide leadership opportunities for students has been acknowledged earlier [38].

**Table 3:** Do’s and Don’ts in starting a LC-SRC

Starting a LC-SRC—Do’s and Don’ts	
Do	Don’t
+ Involve students from different college years and faculty (physicians) +Let students organize the LC-SRC and let them carry the responsibility	- Immediately start an extensive program with many students and doctors. Start a small pilot with only enthusiastic participants and evaluate
+ Try to create additional value for other healthcare workers, patients, and the organization	- Compete for patients with other healthcare workers such as registrars
+ Involve patients (e.g., giving students feedback)	- Underestimate the abilities of motivated students to cope with difficult cases or situations, however provide adequate coaching and supervision

Initial problems encountered in the start-up of the LC-SRC pilot were the time it took to design a LC-SRC (1 year), formulating goals and tasks, and allocating students’ responsibilities. Furthermore, it took some time to recruit medical students, supervisors, and patients. Problems encountered during the pilot were mainly of an organizational nature, such as availability of consultation rooms, access to electronic patient data, and electronic prescribing.

Perhaps the most important lesson learnt in starting a LC-SRC was to identify and provide a solution for a problem in current practice, thereby creating a win-win venture for all stakeholders. Adjacent to education, do try to create additional value for other healthcare workers, patients, and the organization (e.g., starting consultations for therapeutic problems not earlier addressed for reasons such as time constraints or lack of personal). Additionally, this ensures your LC-SRC does not compete for patients with other healthcare workers, such as registrars.

### Limitations to our study

Our study had some limitations, since it was conducted in one outpatient clinic in a single center, with a limited number of participants. The students were selected by application and are therefore possibly not representative of the average medical student. Taking into account these limitations, this study was necessary in order to further develop this concept and determine if scaling up is justified. We have currently extended the project to include three other initiatives all based on the same concept, these new projects are: a student-run cardiovascular risk management program, carried out in a general practitioner’s office, a student-led management and treatment of thyroid diseases, and student-led assessment of adverse drug reaction reports in collaboration with The Netherlands Pharmacovigilance Centre Lareb [39]. The effects of learning in this context should be evaluated in a larger and longer study with control groups and in other settings/disciplines such as pharmacovigilance or family medicine.

In our opinion, early clinical exposure with real responsibility for patient care can improve the pharmacotherapy knowledge and clinical skills of students; however, research is needed to prove this hypothesis. Real context-based learning with responsibility for patient care in a LC-SRC is feasible and an opportunity for medical schools to improve their curriculum.

## Electronic supplementary material

Supplemental digital content 1 (DOCX 14 kb)

## Abbreviations

CBL: Context-based learning
SRC: Student-run clinic
LC-SRC: Learner-centered student-run clinic
