# Supplementary material for: A novel approach to teaching pharmacotherapeutics—feasibility of the learner-centered student-run clinic
Source: Eur J Clin Pharmacol. 2015 Aug 14;71(11):1381–7. doi: 10.1007/s00228-015-1916-x (PMC4613888; doi:10.1007/s00228-015-1916-x)
Supplement: Supplementary file 1 — (DOCX 14 kb) [file 228_2015_1916_MOESM1_ESM.docx]

**Supplemental digital content 1**

| **Baseline characteristics of student participants** | **Total No. (%)** |
| --- | --- |
| **Study year**  **1^st^ year**  **3^rd^ year**  **5^th^ year** | 10 (35%)  9 (31%)  10 (35%) |
| **Gender (Male/Female)**  **Female** | 24 (83%) |
| **How were you selected for medical education**  **Numerus fixus (weigted lottery)**  **Decentralized**  **Cum laude, 8+**  **Lateral entry, from biomedical faculty** | 10 (35%)  15 (52%)  3 (10%)  1 (3%) |
| **Earlier education (in University) (Yes/No)**  **Yes** | 17 (59%) |
| **Extracurricular activities (Yes/No)**  **Yes** | 15 (52%) |
| **Study delay (Yes/No)**  **Yes** | 6 (21%) |
| **Earlier experience in health care (Yes/No)**  **Yes** | 19 (66%) |

Supplemental digital content 1: baseline characteristics of student-participants (n=29 respondents)
